# Emergence and spatial distribution of knockdown resistance in a suburban population of *Aedes albopictus*

**DOI:** 10.1186/s13071-026-07519-6

**Published:** 2026-06-27

**Authors:** Jennifer F. Baltzegar, Cole D. Butler, Jessica Y. Ding, Chay M. Beeson, E. M. X. Reed, Michael H. Reiskind, Martha O. Burford Reiskind

**Affiliations:** 1 Augusta University, Augusta, GA USA; 2 North Carolina State University, Raleigh, NC USA; 3 University of Georgia, Athens, GA USA

**Keywords:** *Aedes albopictus*, Insecticide resistance, Pyrethrins, Mutation, Suburban health, Pest control, Sodium channels, Selection, Geographic mapping

## Abstract

**Background:**

*Aedes albopictus* is a major vector of arboviral diseases and is often targeted by pyrethroid-based mosquito control in residential areas. While knockdown resistance (*kdr*) mutations are well-documented in *Ae. aegypti*, their emergence in *Ae. albopictus* has been less studied, particularly in suburban environments, where insecticide application is often uncoordinated. Understanding the temporal and spatial dynamics of resistance evolution in this context is critical for preserving the effectiveness of public health interventions.

**Methods:**

We conducted longitudinal sampling of *Ae. albopictus* populations in Wake County, North Carolina, from 2016 to 2024. Using a novel allele-specific PCR melt curve assay, we genotyped 2,669 mosquitoes at the F1534S locus in the voltage-gated sodium channel gene. Resistance allele frequencies were calculated annually and mapped across the county for three key years, representing the pre-emergence (2016), initial detection (2018), and widespread phases of resistance development (2023). Selection and dominance were estimated using the Wright–Fisher approximate Bayesian computation algorithm for locus F1534S.

**Results:**

The F1534S resistance allele was first detected in 2018 at a central neighborhood in Wake County. By 2023, the allele had become distributed throughout the sampling region, with the highest observed frequencies near the site of first detection. Resistance allele frequency peaked at 0.36 in 2023, accompanied by an increase in heterozygous and homozygous resistance genotypes. Temporally sampled sites showed consistent trends in rising frequencies of the resistance allele, with all temporally sampled locations harboring resistance genotypes by 2022. The resistance allele was estimated to have a high selection coefficient and to be partially recessive in this population.

**Conclusions:**

Our findings reveal rapid emergence and spatial distribution of the F1534S *kdr* allele in a suburban population of *Ae. albopictus*. The observed distribution of resistance alleles is consistent with strong genetic selection. These results highlight the need for proactive resistance monitoring and integrated management strategies that address private-sector contributions to insecticide selection pressure.

**Graphical Abstract:**

**Figure:**
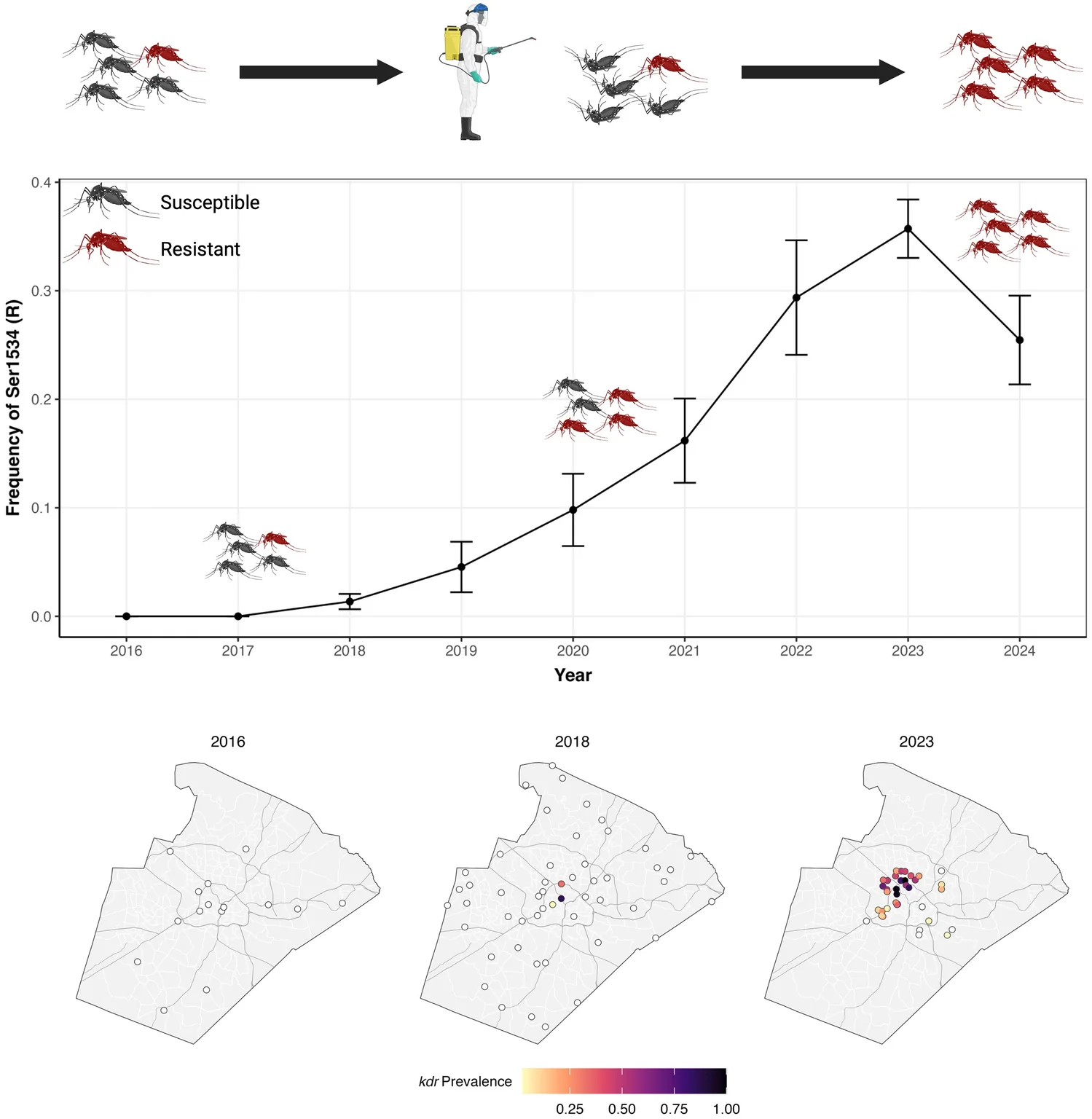

**Supplementary Information:**

The online version contains supplementary material available at https://doi.org/10.1186/s13071-026-07519-6.

## Background

Insecticide resistance is a global public health challenge and a major threat to the control of vector-borne diseases[1]. In mosquitoes, pyrethroids are the most widely used class of insecticides as their relatively low cost and low mammalian toxicity makes them one of the safest options for use in and around homes [2, 3]. With continued use, resistance to pyrethroids can evolve rapidly, undermining the efficacy of these compounds and compromising both epidemic response and nuisance mosquito control. It is critical that best practices for insecticide resistance management are informed by an understanding of how resistance emerges and spreads, particularly in systems, such as those found across much of the United States, where centralized vector control programs are limited or absent.

*Aedes albopictus*, a mosquito species native to Asia, now inhabits every continent except Antarctica and Australia. Its expanding range is fueled by globalization, trade, and a warming climate that opens new habitats for this temperate-adapted species [4]. *Ae. albopictus* is a competent vector for dengue, chikungunya, Zika, and other arboviruses, and is a major vector of dog heartworm [4]. In addition to its role in disease transmission, it is a nuisance mosquito, particularly in suburban areas, where its aggressive daytime biting drives high demand for control [5].

In the United States, efforts to control *Ae. albopictus* are often decentralized. In many suburban settings, including Wake County, North Carolina, mosquito control decisions are typically determined by homeowners rather than through publicly funded municipal programs. In the absence of coordinated public health infrastructure, homeowners contract private companies that offer seasonal pyrethroid-based adulticide treatments often with repeated applications. Previous work has shown that insecticide use can vary with socioeconomic factors and contribute to heterogeneous allele frequency distributions [6, 7]. This uncoordinated approach may, therefore, create localized and repeated selection pressure for resistance to build yet rarely includes resistance monitoring or systematic management practices.

Sustained pyrethroid exposure can lead to strong phenotypic resistance in mosquito populations. Mutations in the voltage-gated sodium channel (*vgsc*) gene, the primary target of pyrethroids, are a well-documented mechanism of knockdown resistance (*kdr*) and can evolve rapidly in response to insecticide pressure [8]. While most studies of *kdr* mutations in *Ae. albopictus* have focused on urban environments with centralized control [9–12], suburban landscapes present a unique opportunity to study resistance evolution in a fragmented, free-market system. In these settings, variation in application frequency, treatment timing, and coverage likely leads to heterogeneous selection pressures across the landscape.

Non-synonymous mutations at amino acid position 1534 in the *vgsc* gene have previously been identified as a key marker of pyrethroid resistance in *Ae. albopictus* (F1534S) and *Ae. aegypti* (F1534C) [9–12]. In both species, point mutations at this locus often represent the first step in the evolution of pyrethroid resistance [8, 9]. While additional mechanisms such as metabolic detoxification contribute to the complete pyrethroid resistance phenotype, Ser1534 has been associated with reduced sensitivity to pyrethroids [12, 13]. It was first identified in populations from China and has now been detected in *Ae. albopictus* populations on multiple continents, including in the United States [11]. However, its emergence and distribution have not been studied in the context of a suburban environment characterized by variable insecticide exposure.

In this study, we develop a high-throughput, cost-effective allele-specific PCR (AS-PCR) assay to rapidly genotype individual *Ae. albopictus* mosquitoes at the F1534S locus. We apply this assay to mosquito collections spanning 9 years (2016–2024) across Wake County, North Carolina, to track temporal and spatial patterns of the Ser1534 resistance allele. With our temporal sampling, we also evaluate the selection pressure in this environment and assess the dominance of the resistance allele. Our goal is to document how quickly the resistance allele increases in frequency in a decentralized control landscape and provide context for vector control and resistance management.

## Methods

### Sample collections

#### Temporal sampling (five sites)

**Fig. 1:**
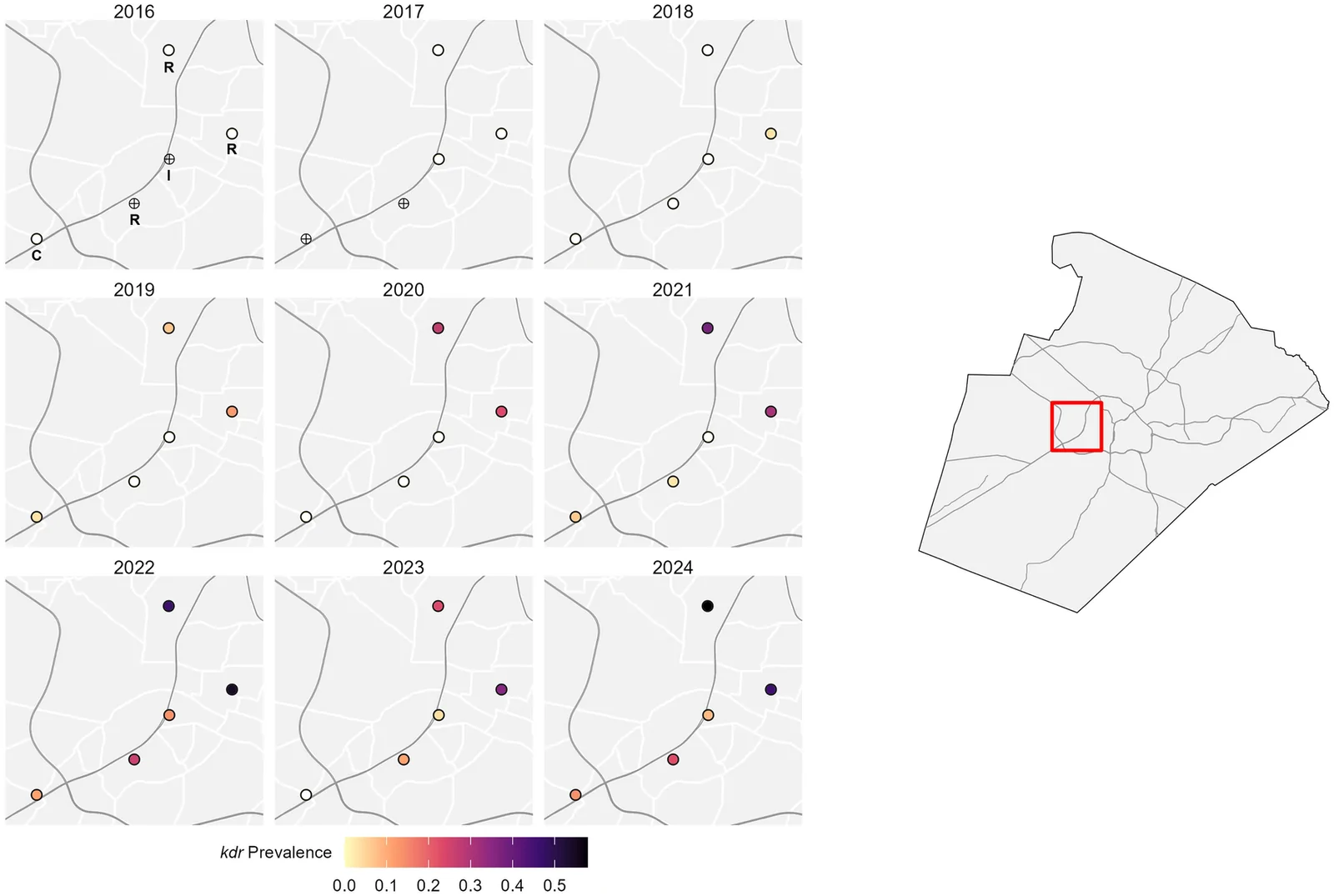
Prevalence of kdr alleles at frequently sampled locations within Wake County. Each circle represents a single sample location (i.e., homeowner's property) within Wake County, North Carolina and circle size represents the sample size at that location. Site property types are labeled in the first panel as R (residential), I (institutional), or C (commercial). Circle color indicates the allele frequency of kdr at that location, with lighter colors representing lower frequencies and darker colors representing higher frequencies. White circles denote locations where mosquitoes were collected but no kdr alleles were detected. White circles with a black cross indicate locations where no sampling was conducted in that year. No mosquitoes contained kdr alleles in 2016 and 2017. Resistance alleles first emerged within these samples in 2018 and quickly rose to very high frequencies at several sites. As of 2024, all five of the frequently sampled locations have moderate to high frequencies of F1534S resistance alleles.

We collected *Ae. albopictus* adults at five sites starting in 2016 and continuing to 2024, as part of ongoing mosquito surveillance in Wake County, NC (Fig. 1). These five sites are located in suburban areas, two are commercial, one is institutional, located at North Carolina State University, and two are at residential homes. We collected adults weekly every summer using BG-Sentinel traps, and in 2016, we used ovitraps to collect eggs [14, 15].

#### Wide spatial sampling (2016, 2018, 2023)

**Fig. 2:**
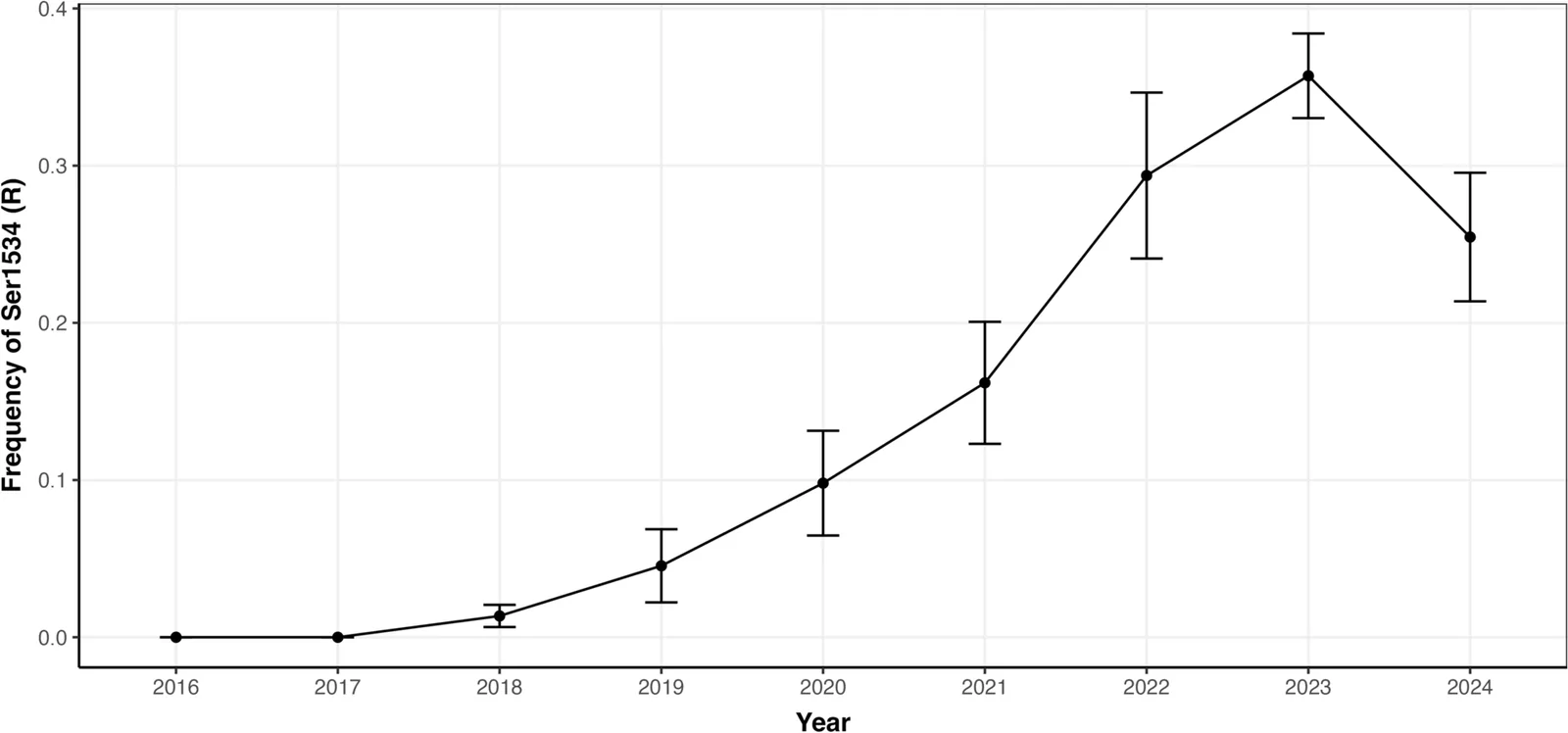
Distribution of kdr alleles in Wake County. Each circle represents a single mosquito collection site within Wake County, North Carolina during years with widespread geographic sampling (2016, 2018, 2023). The color of the circle represents the allele frequency of kdr resistance at that sample location. Lighter colors indicate lower kdr frequencies and darker colors represent higher kdr frequencies. White circles denote locations where mosquitoes were collected but no kdr alleles were detected.

**Fig. 3:**
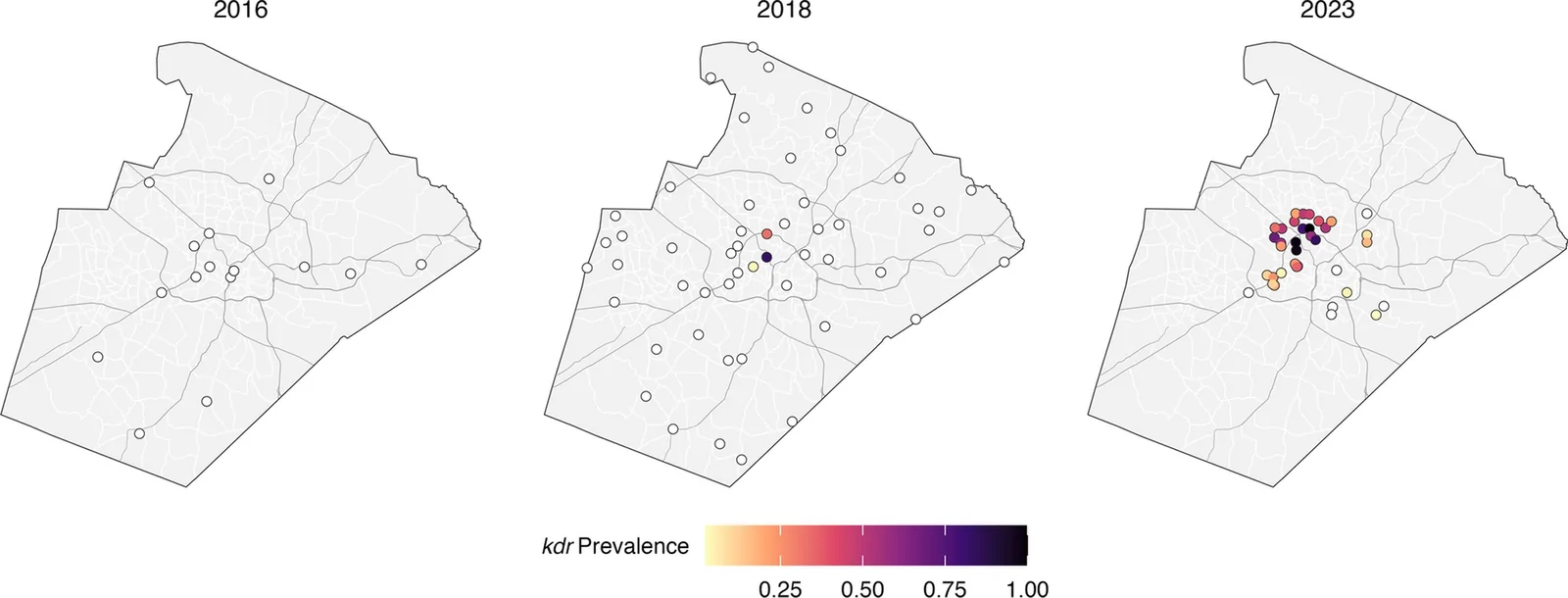
Temporal change in Ser1534 allele frequency from 2016 to 2024. Points represent the annual frequency of the resistance allele across all sampled sites. Error bars indicate 95% confidence intervals. The frequency of the resistance allele increased substantially over the study period with a notable dip in 2024, potentially due to a smaller number of sampled sites in that year.

We conducted spatial sampling for population genetic and ecological studies in 2016 and 2018 that spanned an urban rural gradient. We collected *Ae. albopictus* eggs from sixteen sites in 2016 weekly between 15 April and 26 October 2016 as part of a state-wide mosquito survey effort to characterize the distribution and abundance of *Aedes* species in North Carolina [15, 16] (Fig. [2]). Using ovitraps, we collected eggs which were later hatched, reared, and identified (for full methods, see [16]). In 2018, we collected *Ae. albopictus* adults from 52 sites using BG-Sentinel traps as part of a population genetic study [15] (Fig. 3). We chose the five sites that were part of the annual monitoring program, and the remaining sites were chosen randomly (for full methods, see [15]). These sites spanned an urban to rural gradient in Wake County, NC. Mosquitoes were collected weekly from 15 April to 26 October 2016 and again from 7 June to 25 July 2018, leaving the traps out to collect mosquitoes for 24 h [14, 15]. The larger spatial sampling in 2016 and 2018 allowed us to highlight, where and when Ser1534 first appeared and how the mutation increased its spatial distribution. In 2023, we conducted an a priori*-*designed landscape study at 40 houses from 31 blocks across a wealth and neighborhood age gradient, in addition to the regularly sampled five sites (Fig. 2) [6]. Adult *Ae. albopictus* were collected using BG-Sentinel traps. Samples from all spatial and temporal studies were stored by date and location in a − 80 °C freezer for future processing.

### DNA isolation and quantification

Genomic DNA was extracted from individual mosquitoes using the Qiagen DNeasy Blood and Tissue Kit, with modifications to the manufacturer’s protocol for insect specimens. The head and thorax of each mosquito were homogenized via bead disruption in ATL buffer and proteinase K. Samples were incubated overnight at 56 °C. On the following day, wash steps were performed as prescribed by the protocol, followed by two elution steps in warmed nuclease-free water, yielding a final elution volume of 100 µL. DNA concentration was quantified using a Qubit Fluorometer (ThermoFisher Scientific) with the Qubit 1X dsDNA HS Assay Kit (Cat. No. Q33231).

### Allele-specific PCR genotyping

To genotype individual mosquitoes at the *kdr* locus F1534S, we developed an allele-specific PCR (AS-PCR) melt curve assay based on methods previously used for *Ae. aegypti* [8, 17, 18]. Three primers were designed: one common reverse primer (F1534-Rev: TCTGCTCGTTGAAGTTGTCGAT) and two allele-specific forward primers. The forward primers were designed to uniquely amplify either the susceptible allele (F1534-Fwd: GCGGGCTCTACTTCGTGTTCTTCATCATATT) or the resistance allele (S1534-Fwd: GCGGGCAGGGCGGCGGGGGCGGGGCCTCTACTTCGTGTTCTTCATCATGTC), with GC-rich tags of differing lengths to ensure distinct melting temperatures (~ 4 °C apart) of the resulting PCR products.

Each 10 µL PCR reaction contained: 5.0 µL dH₂O, 0.2 µL S1534-Fwd primer, 0.4 µL F1534-Fwd primer, 0.4 µL F1534-Rev primer, 3.0 µL PerfeCTa SYBR Green SuperMix for iQ (VWR: 101414-144), and 1.0 µL gDNA. Reactions were performed on a Bio-Rad C1000 Touch Thermal Cycler CFX384 Real-Time System (Genomic Sciences Laboratory, North Carolina State University) under the following cycling conditions: initial denaturation at 95 °C for 2 min; 35 cycles of 95 °C for 30 s, 58 °C for 30 s, and 72 °C for 30 s; followed by a final extension at 72 °C for 2 min. A melt curve analysis was then conducted from 65 to 95 °C in 0.2 °C increments every 10 s, with fluorescence measured at each step.

Melting temperature (Tm) peaks were visualized using Bio-Rad Maestro software. The susceptible and resistance alleles produced Tm peaks at approximately 78 °C and 82 °C, respectively. Accordingly, homozygous susceptible individuals showed a single peak at 78 °C, homozygous resistant individuals showed a single peak at 82 °C, and heterozygotes displayed both peaks. To increase confidence in our allele estimates, each plate included genotype controls (SS, SR, and RR) and a no template control. Plates were only scored if all controls performed as expected. In addition, each sample was genotyped in duplicate using the AS-PCR assay, and only those with matching replicate results were included in downstream analyses.

### Validation of the AS-PCR assay

To validate genotype calls from the AS-PCR analysis, we performed targeted probe-capture sequencing of the genomic region containing the F1534S locus using a hybridization-based capture panel (Twist Bioscience, South San Francisco, CA) in a subset of 84 mosquitoes representing all three genotypes (SS, SR, and RR) identified via AS-PCR analysis. Library preparation and target enrichment were conducted following the manufacturer’s protocol and captured libraries were sequenced on an Illumina NextSeq 2000 150 bp paired-end P1 flowcell at the Genomic Sciences Laboratory at North Carolina State University. Genotypes at the F1534S locus were extracted from the resulting variant calls and compared directly to AS-PCR genotype assignments to assess concordance between methods.

### Measures of genetic estimates

Consensus genotypes were determined by comparing results from the two replicate AS-PCR assays for each sample. Samples that failed to amplify in both replicates or produced discordant results were excluded from further analysis. For the remaining samples, genotype and allele frequencies were calculated for each collection year in R using the *tidyverse* package [19, 20]. The selection coefficient and dominance of F1534S were estimated by employing the Wright–Fisher approximate Bayesian calculation (WFABC) model [21]. Because WFABC estimates selection from temporal changes in allele frequency and does not incorporate site-level structure, allele frequencies were aggregated across all samples within each year, and all samples were included despite uneven spatial sampling. In this model, the selection coefficient (s) represents the relative fitness advantage of the resistance allele compared to the susceptible allele, while the dominance coefficient (h) describes the fitness of heterozygotes relative to homozygotes. We assumed a diploid effective population size of 2Ne = 1000, corresponding to Ne = 500 breeding individuals. This value is consistent with effective population size estimates reported for other *Aedes* mosquito populations [22]. Allele frequencies are estimated from the entire population by collection year and number of generations per year is set at 10, which is consistent with *Ae. albopictus* biology in the southeastern USA. Uniform prior distributions were specified for both parameters (min_s = 0, max_s = 1; min_h = 0, max_h = 1), such that s was allowed to vary from 0 (no positive selection) to 1 (maximal positive selection), and h from 0 (fully recessive) to 1 (fully dominant). Posterior samples of s and h generated by WFABC were summarized using marginal distributions and visualized using two-dimensional kernel density estimation in the R package *MASS* to characterize the joint posterior distribution and regions of highest posterior density [23]. All maps were generated using the packages *sf*, *tigris*, *patchwork*, and *tidyverse* in R and included annual allele frequency per site [20, 24–27].

## Results

### Validation of AS-PCR genotyping

High quality sequence data were obtained for 84 samples (Table 1). Concordance between AS-PCR genotypes and sequencing results was high, with 83 of 84 individuals showing matching genotype calls (98.8% agreement; 1.2% error rate). The single discordant sample was classified as heterozygous (SR) by AS-PCR analysis but homozygous susceptible (SS) by sequencing, representing a false positive in the AS-PCR.

**Table 1:** Validation of allele-specific PCR (AS-PCR) genotyping by direct sequencing

Genotype	Years	Mosquitoes	Mismatches
RR	2018	3	0
RR	2023	24	0
RR	2024	2	0
SR	2023	18	1
SS	2016	3	0
SS	2018	26	0
SS	2023	8	0

The table summarizes the number of mosquitoes sequenced for each genotype and year combination, along with the number of mismatches between AS-PCR and sequencing results. Only a single mismatch was observed across all samples (*n* = 1), indicating high concordance and overall accuracy of the AS-PCR assay (98.8% agreement; 1.2% error rate)

### Temporal trends in *kdr* allele frequency

Between 2016 and 2024, we genotyped 2,669 *Ae. albopictus* mosquitoes from 114 unique collection sites in Wake County, North Carolina using allele-specific PCR targeting the F1534S *kdr* mutation (Table 2). Only samples with corroborating genotypes across two technical replicates were retained for analysis (n = 2,273). Resistance alleles were not detected in the first 2 years of the study (2016–2017), but their frequency increased steadily thereafter, reaching a maximum of 0.36 (95% CI ± 0.027) in 2023 (Fig. 3). In 2024, the resistance allele frequency declined to 0.25 (95% CI ± 0.044). Across the same period, the proportion of homozygous susceptible genotypes (SS) decreased, while heterozygous (SR) and homozygous resistant (RR) genotypes increased.

**Table 2:** Temporal changes in allele and genotype frequencies at the F1534S locus in *Aedes albopictus* from Wake County, North Carolina, 2016–2024

Years	Sites (n)	Mosquitoes (n)	Freq. R Allele	Genotype frequencies		
				SS	SR	RR
2016	16	225	0.00	1.00	0.00	0.00
2017	3	82	0.00	1.00	0.00	0.00
2018	52	516	0.01	0.98	0.00	0.01
2019	5	154	0.05	0.93	0.05	0.02
2020	5	153	0.10	0.86	0.08	0.06
2021	5	173	0.16	0.77	0.13	0.10
2022	5	143	0.29	0.61	0.20	0.20
2023	36	609	0.36	0.52	0.25	0.23
2024	5	218	0.25	0.67	0.14	0.18

The table shows the number of collection sites and mosquitoes successfully genotyped each year, the frequency of the resistance (R) allele, and the frequencies of the three genotypes: homozygous susceptible (SS), heterozygous (SR), and homozygous resistant (RR)

### Temporal dynamics of resistance

To track resistance emergence and spatial distribution at consistent locations, we conducted repeated sampling at five fixed sites, within suburban residential areas, from 2016 to 2024. In 2016 and 2017, we only found mosquitoes at three of the five sites and the mosquitoes collected showed only homozygous susceptible genotypes. The F1534S resistance allele first appeared in 2018 at one of the sampled sites. [A nearby second site included in the expanded spatial sampling for 2018 (see below) also tested positive for resistance alleles in 2018 (Figs. 1 and 2)]. By 2022, all five of the temporally sampled sites tested positive for mosquitoes carrying at least one copy of the resistance allele (Fig. 1). By 2024, each site exhibited moderate to high frequencies of Ser1534, indicating a rapid increase in resistance allele frequency since 2016.

### Spatial distribution of resistance

Broader spatial sampling conducted in 2016, 2018, and 2023 captured a wider geographic pattern of *kdr* allele distribution across Wake County. Following a similar pattern to resistance emergence as the temporally sampled sites, all genotyped mosquitoes were homozygous susceptible in 2016. In 2018, the resistance allele was detected at low frequency and localized primarily at two sites near the center of the county (including one of the temporally sampled sites), an area characterized by older and more affluent neighborhoods. By 2023, resistance alleles were found throughout the sampling region, with the highest allele frequencies concentrated near the original 2018 detection zone (Fig. 2). Peripheral areas of the county generally exhibited lower resistance frequencies.

### Genetic parameters of resistance

**Fig. 4:**
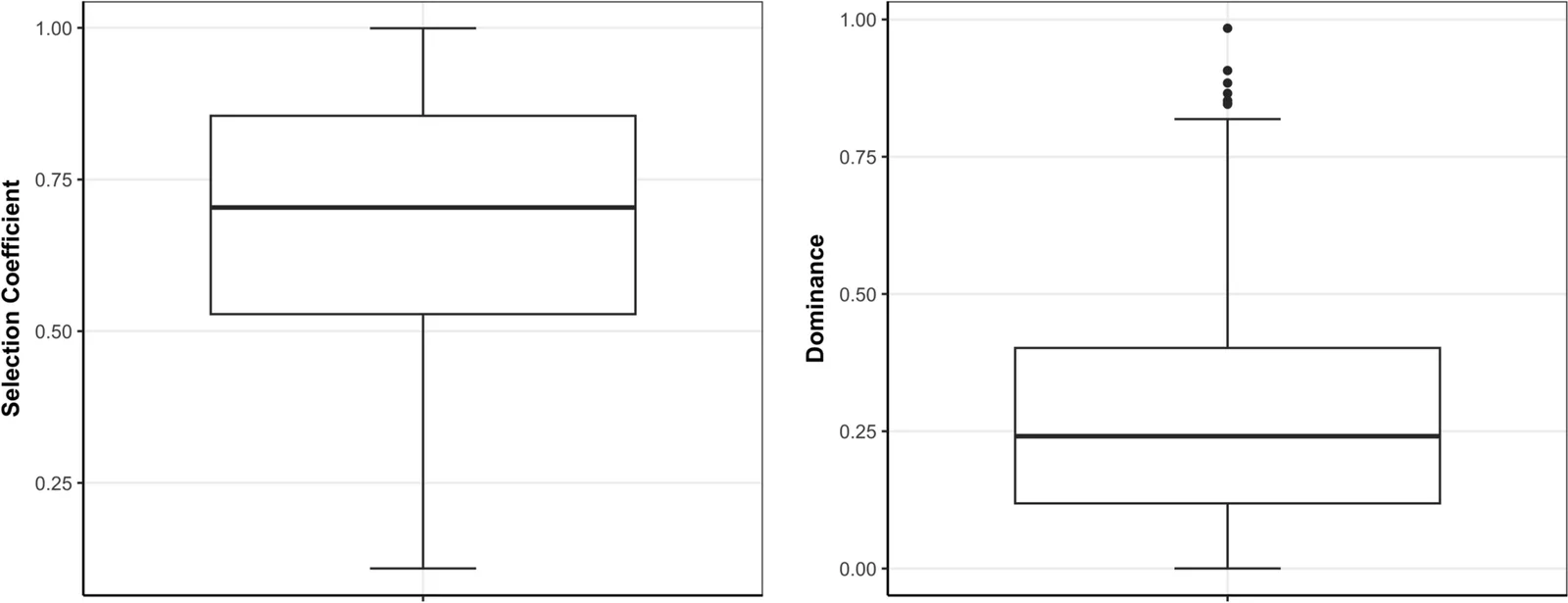
Wright–Fisher approximate Bayesian computation (WFABC) estimates for the F1534S locus. Box plots summarizing the posterior distributions of the selection coefficient (s) and dominance (h) based on 1,000 simulated datasets. The center line denotes the median, lower and upper boxes represent the 1st and 3rd quartile, respectively, and whiskers extend to 1.5 times the interquartile range. Outliers are represented by points beyond the whisker range. Together, these distributions indicate strong positive selection (mean *s* = 0.68) and partial recessivity (mean *h* = 0.27)

**Fig. 5:**
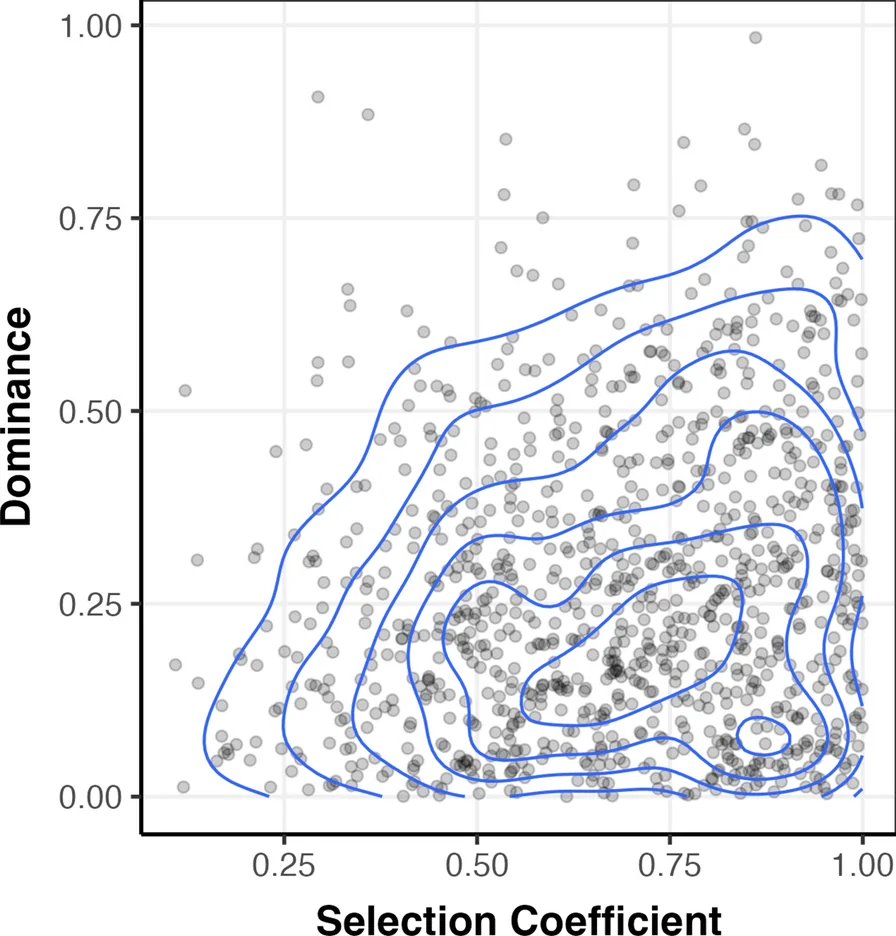
Two-dimensional kernel density plot. Joint posterior distributions of the selection coefficient (s) and dominance (h) inferred using WFABC. Points represent samples from the joint posterior distribution. Blue contours represent constant probability densities with smaller encircled areas representing higher probabilities

Using time series allele frequency data collected from both the temporal and spatial datasets, we estimated the posterior distribution of the selection coefficient (s) and dominance (h) of the F1534S resistance allele using the WFABC algorithm [21]. The mean selection coefficient was 0.68, with a 95% credible interval of 0.25 to 0.99 (2.5th to 97.5th percentile). The mean dominance coefficient was 0.27, with a 95% credible interval of 0.01 to 0.71, indicating that the resistance allele is partially recessive. Box plots summarizing the marginal posterior distributions of s and h (Fig. 4) highlight estimate uncertainty and inter-correlation. However, the two-dimensional kernel density plot of the joint posterior distributions (Fig. 5) supports a region of highest posterior density characterized by strong selection and partial recessivity.

## Discussion

Monitoring insecticide resistance in mosquito populations is critical for maintaining effective vector control strategies. In this study, we provide the first longitudinal dataset documenting the emergence and spatial distribution of the Ser1534 *kdr* allele in *Aedes albopictus* from a suburban environment in the United States. Over the 9-year study period, we observed a clear increase in both the frequency and geographic distribution of this resistance allele in Wake County, North Carolina, consistent with strong and ongoing selection pressure, as supported by the estimated selection coefficient.

Our results indicate that the resistance allele at locus F1534S (i.e., Ser1534) was not detected in local *Ae. albopictus* populations prior to 2018. Following its initial detection, it has risen in frequency across all temporally sampled sites, reaching moderate to high frequencies by 2023. Early detections were concentrated in central Wake County, an area characterized by older, more affluent neighborhoods. By 2023, Ser1534 was present at high frequencies across most sampled sites in this region. Although resistance allele frequency was slightly lower in 2024 compared to 2023, this apparent decline coincided with substantially reduced sampling effort in 2024 (*n* = 5) compared to 2023 (*n* = 36), limiting direct year-to-year comparisons. Future work should maintain consistent spatial coverage across years to better track ongoing dynamics.

While these descriptive spatial patterns do not allow us to definitively infer a point of origin or directional spread, the observed temporal increase and central spatial concentration are consistent with localized selection and subsequent dispersal. Human-mediated insecticide use and local mosquito movement represent plausible mechanisms underlying these patterns, though formal spatio-temporal modeling will be required to distinguish among alternative explanations. Future work should incorporate formal spatial statistical approaches to quantitatively assess the degree of spatial structure and identify potential hotspots of resistance allele frequency.

We hypothesize that residential insecticide use, particularly professionally applied barrier sprays by private pest control companies, is a major driver of the increase in the Ser1534 resistance allele in this system, where municipally coordinated mosquito control is limited. Although initial detections occurred in areas generally characterized as more affluent, socioeconomic variables were not quantified here. Previous studies have linked socioeconomic status, insecticide use, and resistance evolution in *Aedes aegypti* and *Ae. albopictus* [6, 7], suggesting a potential mechanism that warrants direct evaluation in future work. Although *Ae. albopictus* in Wake County has not yet been implicated in major arboviral outbreaks, the increasing risk of diseases such as dengue and chikungunya in the southeastern United States highlights the importance of preserving effective vector control tools [28].

Our results, together with previous work in this population, support the role of Ser1534 as a sentinel allele and early indicator of increasing insecticide resistance [29]. The estimated selection coefficient for Ser1534 was high (*s* = 0.68). This finding is consistent with strong directional selection favoring the resistance allele. However, the posterior distribution indicates uncertainty in the magnitude of this estimate. This pattern aligns with a scenario of strong insecticide-mediated selection, particularly given the assumed widespread use of pyrethroid products in residential settings, although insecticide exposure was not directly quantified in this study.

The allele frequency of Ser1534 is partially recessive (*h* = 0.27) and, therefore, is expected to increase rapidly once homozygotes become common, given that heterozygotes retain partial susceptibility. As with s, the posterior distribution of h indicates uncertainty in the precise estimate, but is broadly consistent with incomplete recessivity. These dynamics have important implications for resistance management. Incomplete recessivity can delay the fixation of resistance alleles. As a result, low-level resistance may persist undetected in the absence of molecular genotyping. This can continue until phenotypic resistance becomes widespread and control failure occurs [30].

Our findings underscore the urgent need for routine resistance monitoring in *Ae. albopictus*, particularly in suburban settings, where private pesticide use is common. Although incorporating genetic surveillance of loci such as F1534S and other *kdr* mutations into routine operations would be ideal, most vector control programs in the United States lack the technical capacity and resources to implement such monitoring. Recent assessments of publicly funded mosquito control capacity in Florida and Texas indicate that only a minority of jurisdictions are fully capable of meeting core operational standards [31]. Many programs report insufficient funding, limited personnel, and inadequate surveillance infrastructure as reasons for a lack of monitoring. Importantly, the availability of funding and technical support likely differs between publicly and privately operated vector control programs, further influencing their ability to adopt genetic surveillance methodologies. Given these constraints, sustained support from state and federal agencies will be essential to expand genetic surveillance capacity and maintain the long-term efficacy of insecticides. Furthermore, public health authorities and pest control companies should work collaboratively to improve and promote integrated vector management strategies that slow the spread of phenotypic resistance to insecticides.

## Conclusions

This study documents the rapid emergence and observed spatial distribution of the Ser1534 *kdr* resistance allele in *Aedes albopictus* across Wake County, North Carolina over a 9-year period. The resistance allele was first detected in 2018 and has since increased in frequency and geographic extent, consistent with the strong positive selection coefficient estimated in this study. While these patterns are consistent with selection pressure, including potential contributions from suburban residential use of pyrethroid-based insecticides, the specific drivers of selection were not directly evaluated. Given the persistence and potential public health impact of this mutation, we recommend routine monitoring of *kdr* alleles in *Ae. albopictus* and greater collaboration between public health agencies and private pest control services to implement improved insecticide resistance management strategies.

## Supplementary Information

Supplementary Material 1.

## Data Availability

The datasets generated and analyzed during the current study are available in the DRYAD repository, https://doi.org/10.5061/dryad.t76hdr8ct.

## Abbreviations

*Ae. albopictus*: *Aedes albopictus*
*Ae. aegypti*: *Aedes aegypti*
AS-PCR: Allele specific polymerase chain reaction
D: Disequilibrium
h: Dominance
HWE: Hardy–Weinberg equilibrium
*kdr*: Knockdown resistance
Ne: Effective population size
RR: Homozygous resistance
s: Selection coefficient
SR: Heterozygous
SS: Homozygous susceptible
Tm: Melting temperature
*vgsc*: Voltage-gated sodium channel
WFABC: Wright–Fisher approximate Bayesian calculation
